# National Voice Campaign - 2012

**DOI:** 10.5935/1808-8694.20130122

**Published:** 2015-10-08

**Authors:** Gustavo Korn

**Affiliations:** Department of Otorhinolaryngology and Head and Neck Surgery, Federal University of São Paulo. Director of the Brazilian Academy of Laryngology and Voice (ABLV) and National Coordinator of the Voice Campaign 2012–2013

The 14^th^ National Voice Campaign, held between the 16^th^ and 20^th^ of April, 2012, involved the “big throat” exhibit in the Ibirapuera Park (São Paulo), when we also provided medical care to the population.

The Campaign numbers were really exciting. In the inflatable “big throat” exhibit we had over 70 residents from different services and over 10,000 visitors. As far as the medical care provided is concerned, we had over 80 public and private accredited services from all over the country participating in the event. It is worth noting that almost a third of the care provided was held at the teaching hospital of the Medical School of the University of São Paulo (FMUSP), with great help from Dr. Adriana Hachiya (ABLV Director of the Brazilian Academy of Laryngology and Voice and Local Coordinator of the Voice Campaign).

Through information obtained from 1,800 medical care forms filled out, we prepared the descriptive analysis presented below. To compare each of the study findings in relation to gender and age we used the chi-square test and, when needed, Fisher's exact test.

Most of the care provided was in the Southeast (64.2% of 1,399), followed by Northern Brazil (18.4% of 1,399) and the Northeast (12.5% of 1,399). The State of São Paulo had 61.5 % of the visits. There was a predominance of females (67.6% of 1,784), and the mean age was 48.6 years (standard deviation of 16.7 years of 1,692). Among the complaints, we had hoarseness/voice changes (15.4%), dysphagia (9.4%), hawking (4.1%), sore throat (3.6%), other complaints (5.3%); and 50.4% of the sample had more than one complaint. Only 11.7% had no complaints. Regarding symptom duration of 1,203, most had had symptoms for more than one year (53.9%), 13.9% lasted between 6 months and 1 year, and 27.6% between 15 days and 6 months. Only 4.7% had less than 15 days of complaint. The majority of the sample was comprised of non-smokers (61.5% of 1,151) and did not drink alcoholic beverages (70.1% of 857). Almost a third of the sample reported using their voices professionally (36.1% of 1,358).

Larynx examination was performed in 1,598 patients. Reflux was the main finding (40.9%), followed by vocal fold nodules (6.8%), minor structural changes (4.9%), Reinke's edema (4.3%), and polyps (3%). Tumor was suspected in 1.6% of the sample. A normal laryngeal exam, except minor structural changes, was observed in 28.54%.

Comparing the data, we observed a predominance of vocal fold nodules, Reinke's edema, and vocal cysts in females, while males had a predominance of leukoplakia and tumors. Nodules, polyps, cysts and minimal structural changes predominated in the age group up to 60 years, and leukoplakia, paralysis, and tumors predominated in those aged over 60 years.

Attention should be paid to reflux, found in more than a third of our sample. It is also worth stressing the suspected tumors in 1.6% of the sample, i.e. 25 patients. One of the main concerns of the Voice Campaign is to find laryngeal cancer, at an early stage, if possible. In this study, these patients we suspected could be screened and were targeted for further assessment and treatment.

We must stress three limitations to this study:

1. Most of the patients seen had some vocal complaint (approximately 88%);
2. Many patients did not filled out the entire form;
3. The systematic evaluation was not uniform (some colleagues used nasofibrolaryngoscopy, others used rigid scope telelaryngoscopy and some used stroboscopy).

This is the first nationwide survey of the Voice Campaign and this data is of great importance to the Brazilian Academy of Laryngology and Voice and to the Brazilian Association of Otorhinolaryngology and Neck and Facial Surgery (ABORL-CCF) to use when pleading support from the Ministry of Health. We hope to repeat this study in the future and to be able to count on the valuable and most kind help from a larger number of colleagues. Similarly, this survey can serve as a basis for a broader national or international epidemiological research project.

Finally, I would like to extend my gratitude to all who helped in this campaign - doctors and clinics that participated in the exhibition at Ibirapuera park, and those who participated in patient care; the entire ABORL-CCF staff, people from Sintonia; the ABLV Board, led by Dr. José Eduardo de Sá Pedroso.

